# An Emulsion Based Microarray Method to Detect the Toxin Genes of Toxin-Producing Organisms

**DOI:** 10.3390/molecules16097365

**Published:** 2011-08-29

**Authors:** Ying Wang, Jiafeng Lu, Qi Yang, Yunfei Bai, Qinyu Ge

**Affiliations:** 1 Key Laboratory of Child Development and Learning Science, Ministry of Education, Southeast University, Nanjing 210096, China; 2 State Key Laboratory of Bioelectronics, Southeast University, Nanjing 210096, China

**Keywords:** emulsion PCR, microarray, toxins

## Abstract

Toxins produced by bacteria and fungi are one of the most important factors which may cause food contamination. The study of detection methods with high sensitivity and throughput is significant for the protection of food safety. In the present study, we coupled microarray with emulsion PCR and developed a high throughput detection method. Thirteen different gene sites which encode the common toxins of several bacteria and fungi were assayed in parallel in positive and maize samples. Conventional PCR assays were carried out for comparison. The results showed that the developed microarray method had high specificity and sensitivity. Two zearalenone-related genes were investigated in one of the ten maize samples obtained with this present method. The results indicated that the emulsion based microarray detection method was developed successfully and suggested its potential application in multiple gene site detection.

## 1. Introduction

Recently, food safety has become more and more of concern due to reported problems such as contaminated ham and cucumber. Toxins, pesticide residues and many other factors might contribute mainly to this problem [1,2,3]. Among them, contamination in food or food sources by microbial toxins was one of the most important aspects of food safety. Hence, the availability of reliable and efficient detection methods for these toxins is very important; the conventional methods for the detection of toxin-producing microorganism such as fungi are chemical analysis, instrumental analysis, immunoassay, and bacterial luminescent tests, *etc*. [2,3,4,5,6,7]. However, low sensitivity, poor reproducibility, long duration and poor security are the common problems in these methods. Moreover, there is no stable method that can simultaneously detect the most common microbial toxins. It is very significant to establish an accurate and efficient method which could detect toxin-producing fungi, bacteria and other microorganisms in a high-throughput manner.

Microarrays have provided a platform for high-throughput detection, and have been successfully used for mRNA expression analysis [8,9,10], mutation analysis [11], DNA methylation analysis and deletion strain analysis [12]. However, the widely application of DNA microarrays in microorganism detection was limited by its target DNA preparation method such as multiplex PCR. The interferences between primers and templates might be the main reason for the poor results [13,14], so it is necessary to study it further.

Emulsion PCR was developed in the last decade and is widely used in many aspects such as the next generation DNA sequencing technology [15,16,17,18]. We have successfully developed an emulsion based multiplex PCR using separated micro-droplets [19]. However, the limited number of primers has restrained its ability of parallel amplification, and only products of different length could be separated by electrophoresis. These problems could be solved by DNA microarrays, which have no requirement for different amplification products length with different genes and the sequencing information could be confirmed by hybridization, which highly increases the reliability of the method, as well as the number of detection sites is also limitless for DNA microarrays. 

BEAMing was a system whose amplification was carried out on the surface of magnetic beads in emulsion [20,21,22,23]. It had been used in commercial sequencing platforms such as SOLiD and the 454 sequencing system for its high throughput potential [24,25]. Compared with conventional emulsion PCR, BEAMing had higher efficiency with relative low concentrations of primers and good separation of the micro-droplets by introducing the magnetic beads. 

In present study, the side products of BEAMing were collected and labeled with fluorescence, then detected by DNA microarray. This BEAMing-coupled DNA microarray method was developed and validated by using to inspect fungi and bacteria which could produce toxins. The results showed that the desired gene sites were detected by the combined method specifically and sensitively, and it also revealed good application prospects.

## 2. Results and Discussion

### 2.1. Comparison of Immobilization Approaches for Primer on Beads Surface

**Figure 1:**
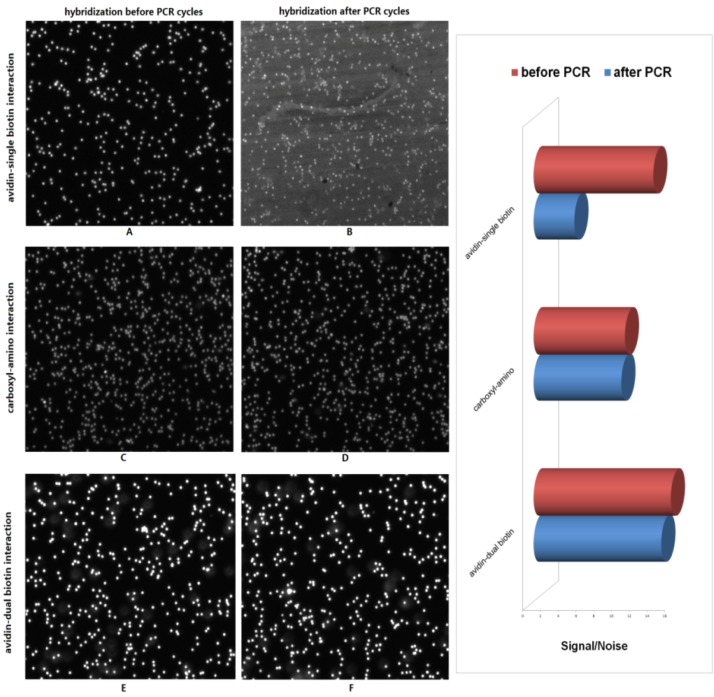
Comparison of different immobilization methods. Three different interactions between primers and the beads surface are shown. Pictures A/B, C and D; E and F show the hybridization signals before and after PCR thermal cycle of avidin-biotin interaction, carboxyl-amino interaction and avidin-dual biotin interaction, respectively; histograms (right) reveal the clear differences of the three immobilization method applied in emulsion PCR.

The immobilized efficiency of these different primers on the beads was very important to BEAMing, especially in this study, because the target molecules for hybridization on the DNA microarray were provided by effective amplification on the bead surface. Three different methods for primer immobilization were compared. The results are shown in Figure 1, where the hybridized signals on beads were captured by a CCD camera under a fluorescent microscope (Nikon E300). A, C and E in Figure 1 show the pictures captured before PCR thermal cycles with avidin-biotin, carboxyl-amino and avidin-dual biotin interaction, respectively. Satisfactory signal/noise ratios were obtained by all three methods. However, a remarkable decrease of the signal/noise ratio was found in avidin-biotin interaction after the PCR cycle (picture B); no obvious differences were found between the other two methods (pictures D, F). We finally selected the avidin-dual biotin immobilization method for its higher signal/noise ratio compared to the method with carboxyl-amino interaction.

### 2.2. Reliability and Sensitivity of the BEAMing Based Method

**Figure 2:**
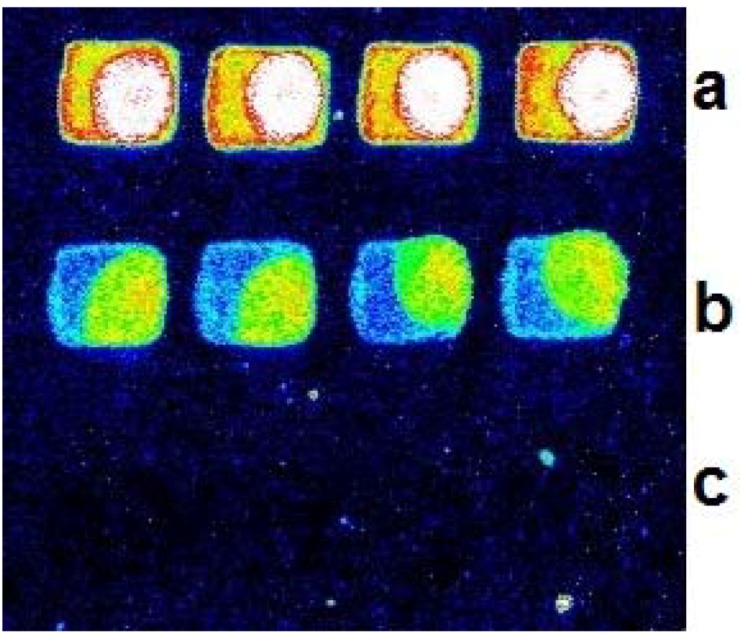
Specificity of the beads emulsion based detection method. The figure shows one typical hybridization picture of the BEAMing based method with positive and negative control; (**a**) hybridization signal of SRY gene from male DNA which act as positive control; (**b**) signal of one toxin gene of pksA from micrograms DNA; (**c**) an unrelated gene used in the study as negative control.

Then we studied the reliability of the BEAMing based microarray method. One male specific gene (SRY) was spotted on the slide as positive control and another unrelated oligo was also spotted alternately on the slide as a negative control. pksA gene was selected as an example for study. One male genome DNA and the DNA extracted from microgram were mixed for detection. Four repeats were spotted in each row. The results are shown in Figure 2, where no signal was found in the negative control spots.

A 10× serial dilution of male DNA templates quantified previously were used to evaluate the sensitivity of the BEAMing based method compared with conventional PCR. 

**Figure 3:**
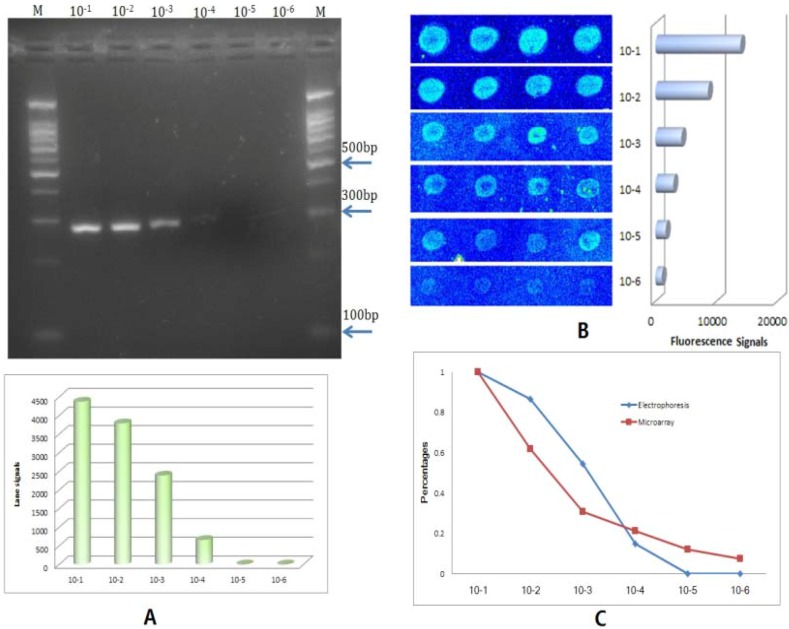
Comparison with conventional PCR. (**A**) Electrophoresis photo of 10×serial dilution of male DNA detected conventional PCR; a clear band could be identified in 10^−3^ of the template DNA, and a weak band at 10^−4^ dilution; the bottom histogram denotes the result analyzed by a Gel Imaging System (BioRad); (**B**) the same detection result of the BEAMing based method, showing positive signals in 10^−5^ dilution and a weak positive signal even at 10^−6^ dilution; (**C**) signal comparison of the two methods, which shows their sensitivity differences.

We can see in Figure 3 that the conventional PCR results showed that it could detect as little as 1 × 10^−4^ of the template DNA (5 ng/μL of template DNA, Figure 3A) and that a positive result was also obtained with 1 × 10^−4^ of template DNA by the BEAMing based microarray method (Figure 3B), while weak but identifiable signals were also seen at 1 × 10^−5^ (500 pg/μL of template DNA) by this coupled method. It could be seen in this study (Figure 3C) that hybridization based detection method showed more sensitivity than gel electrophoresis.

### 2.3. Detection of the Toxins Producing Bacteria with BEAMing Based Microarray Method

**Figure 4:**
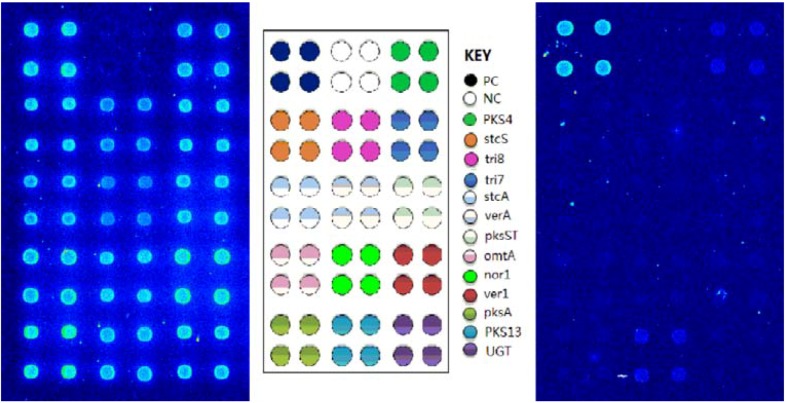
Detection of toxins gene with the BEAMing based microarray method: A is one typical detection result of whole positive DNA by the BEAMing method, it showed that all the gene sites except negative control obtained clear hybridization signal; B shows the location of the 13 gene sites and control gene in the microarray; C shows that only one maize sample gave a positive signal in this validation study, the two zearalenone-related genes showed relative weak but clear positive signals in the picture.

In the present study, 13 gene sites from different bacteria and fungi were selected and amplified simultaneously by emulsion-based multiplex PCR, and then detected by DNA microarray. The extracted positive DNA were mixed and detected by this method, results showed that corresponding positive signals were all detected (Figure 4A), the delineation of gene sites positions on the microarray are shown in Figure 4B. Ten maize samples were purchased from different shops for investigation in this study, genome DNA were extracted and detected by the proposed method. Results showed that two gene sites, PSK4 and PKS13, gave weak positive signals in only one of the samples (Figure 4C).

Multiplex PCR is the most commonly used method in these related studies of microbiology detection [26,27], while its parallel amplification ability was largely limited by the interferences between primers. Currently, no method can solve this problem thoroughly, although many attempts have been made [28,29]. In present study, we coupled BEAMing with DNA microarrays to develop a multiplex detection method, and thirteen related toxins gene sites from toxin-producing organisms were simultaneously investigated successfully. These achievements in the method mainly contribute to the following aspects: (1) BEAMing beads which were coated with one of the pair primers: different primers were separated by the beads in different emulsion droplets; the concentrations of the primers were also reduced highly in the reaction and this resulted in less interference between primers. (2) PCR amplification in emulsion system: thousands upon thousands of micro-droplet in emulsions mostly separate the different gene amplification. Just because of this, emulsion PCR was used to produce monoclonal sequencing templates in the next generation sequencing technology. (3) DNA microarray was used for final detection: high throughput is the primary concern for microarrays. In this study, the microarray was mainly used to replace electrophoresis against multiplex gene sites detection. It showed more flexible and reliable results without length limitation and sequence based detection. The hybridization based microarray method used in this study also showed better sensitivity than conventional PCR with electrophoresis (Figure 3C). Beads-based amplification might contribute to this mostly, because all the target molecules for hybridization were single strand DNA obtained from amplified magnetic beads. All these factors mentioned above might contribute to the success and also suggested a reliable, sensitive and high throughput detection method.

High efficiency of immobilized primers on magnetic beads was the key factor for a successful emulsion PCR. The different methods for immobilizing oligos on beads have been mentioned by Dressman *et al*. in their research [30]; however, systematic and general study of the common approaches in this study was more significant. Carboxyl-amino interaction on the bead surface could also be used in the method for much cheaper modification of the primer by amino than dual biotin, although we selected dual biotin in present study for the possibility of producing more PCR products.

Two related toxins gene were detected in this study. We can infer from the result that the one maize sample detected were probably contaminated by zearalenone produced by *F. graminearum*, because this toxin was coded mainly by the two genes PKS13 and PKS4 detected in the sample [31].

## 3. Experimental

### 3.1. Sample Preparation

Ten maize samples used as the raw material for animal feed were purchased for the study from different grain shops. DNA was extracted according to Holden’s report with some modifications [32]. Positive DNA samples used in this study were obtained as a gift from the Food Science and Technology College of Hunan Agriculture University. One male DNA was used as positive control in the study which was mixed with the sample before emulsion PCR.

### 3.2.Preparation of the Oligonucleotides and Microarray

Aminosilane-derivatized glass slides were cleaned with deionized distilled water and incubated in 5% glutaraldehyde in 0.1 mol/L PBS, pH 7.4, for 2 h. Then, the slides were thoroughly washed twice with methanol, acetone, and deionized distilled water and dried for use. Two hours after the probes were spotted on the treated glass slides, the slides were incubated in 0.01 mol/L NaBH_4_ solutions for about 30 min, then they were thoroughly washed twice with distilled water and dried for use. The probes and primers of the used gene sites in this study were synthesized and purified by Invitrogen Inc. (Shanghai, China). Cy3-dUTP was purchased from Amersham (GE Healthcare) for target DNA labeling. The DNA sequences and GenBank number of probes and primers are listed in Table 1.

**Table 1:** DNA sequence of the primers and probes.

Gene name		Primers/Probes sequence (5’-3’)	Modification	GenBank No.	Toxins
PKS4		(F)gtatcttggagctgccttgc		gi:46562332	Zearalenone
		(R)ccaccacccaaaagcttaaa			
		(P)ccatcggcactaggagacat	5’-amino		
stcS		(F)ggtggtggagctgtgaatct		gi:50058538	Sterigmatocystin
		(R)ccgatgaggtcgttgttttt			
		(P)gttcctgttctggccttctg	5’-amino		
Tri8		(F)tttgctggaacttgtgttgc		gi:4249355	Trichothecene
		(R)gtatacagcgccacctggat			
		(P)ctagtcaagtttccaggcgc	5’-amino		
Tri7		(F)tgtttgcctcatcttcaacg		gi:4249355	Trichothecene
		(R)acattgccacgcaacaataa			
		(P)agcaaagcatctttgtggct	5’-amino		
stcA		(F)ggtggaacatgacacactgc		gi:50058538	Sterigmatocystin
		(R)gctacgtcttgggagtctgc			
		(P)atttcaaggttatcgcgcac	5’-amino		
verA		(F)tatggcctgtccctatctcg		gi:50058538	Sterigmatocystin
		(R)gctgtccaggaggtgaagag			
		(P)tgctgtcctccaaccatgta	5’-amino		
pksST		(F)gctacgtcttgggagtctgc		gi:50058538	Sterigmatocystin
		(R)ggtggaacatgacacactgc			
		(P)gtgcgcgataaccttgaaat	5’-amino		
omtA		(F)ctcctctaccagtggcttcg		gi:169775554	Aflatoxin
		(R)aacctccgagttggaatgtg			
		(P)ccgcccatacctagatcaaa	5’-amino		
Nor1		(F)cacttagccagcacgatcaa		gi:169775554	Aflatoxin
		(R)tttgggacgttggagaaaag			
		(P)ccgaggtacggtctatcgaa	5’-amino		
Ver1		(F)tccccaatggtgagactttc		gi:169775554	Aflatoxin
		(R)caccccaatgatctttccac			
		(P)ccccataaactgcgtcttgt	5’-amino		
pksA		(F)gaacgtaccggatgaagcat		gi:169775554	Aflatoxin
		(R)atgctgcagagcatgaacac			
		(P)gaggcacactagagcggttc	5’-amino		
PKS13		(F)tgggcgcttaagactgagat		gi:46562332	Zearalenone
		(R)atttccccaccaaacatgaa			
		(P)ttgaatcctggatccgaaag	5’-amino		
UGT		(F)acgagaagctgatcgtggac		gi:21240774	Deoxynivalenol
		(R)ttacatgccagagccttcct			
		(P)gttgaaggggaggacatgag	5’-amino		
SRY		(F)acctgttgtccagttgcact		gi:4507224	--
		(R)actgaaagctgtaactctaagta			
		(P)tgaagcgacccatgaacgcattca	5’-amino		

(F): Forward primer; (R): Reverse primer; (P): Probe.

### 3.3. Strategy of the Emulsion Based Detection Method

**Figure 5:**
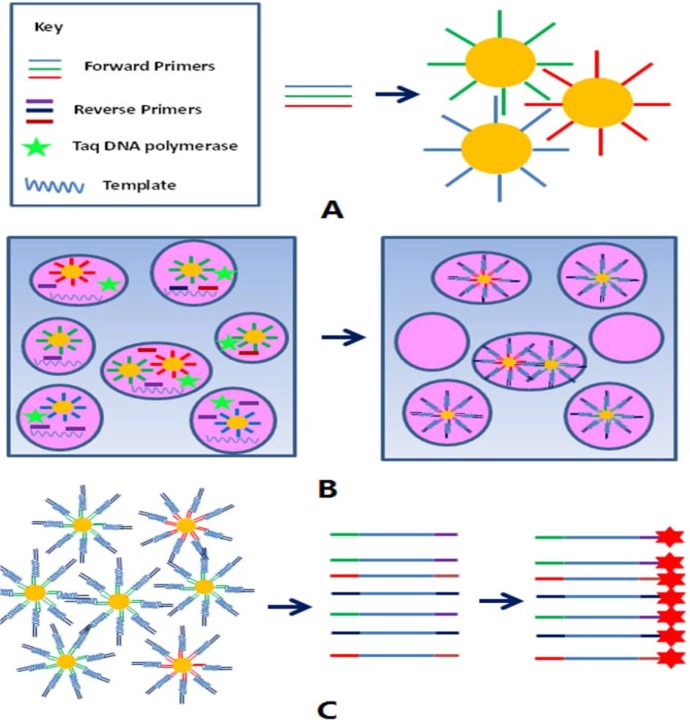
Main pipeline of the BEAMing based microarray detection method: (**A**) shows different primers that were immobilized on the carboxyl-coated beads respectively, and mixed before BEAMing; picture (**B**) denotes the possible condition of the emulsion system before and after PCR thermal cycles. Product amplification occurred only in the micro-droplet which contained the corresponding primers and other PCR components; (**C**) in the figure shows that the desired single strand DNA for microarray hybridization could be separated from the amplified beads easily and be labeled with Cy3 fluorescence by terminal transferase.

Figure 5 is a schematic diagram illustrating the BEAMing based microarray detection method. The forward primers of all the gene sites inspected were immobilized on the beads surface, and then balance mixed for emulsion PCR. 

An emulsion system containing these forward primer coated beads, all the reverse primers and other PCR components were prepared; only those micro-droplets which contain DNA polymerase, template DNA, and the corresponding reverse primers could generate PCR products. After the PCR thermal cycles, the emulsions were broken and the amplified beads were pooled. Most of the washed beads were well amplified and contained double strand PCR products in the surface, the free stranded DNA products were separated from the beads by 0.1M NaOH which acted as denaturing buffer, then purified with a MinElute® Reaction Cleanup Kit (QIAGEN).

All the 3’-end of the single strand PCR products obtained were labeled with Cy3 fluorescence by terminal transferase, and then hybridized with the probes previously immobilized on the slides. The hybridization signal of the spots could be obtained by a laser scanner which indicated the positive or negative result for the corresponding detected gene site.

### 3.4. BEAMing

Magnetic beads of 1.05 ± 0.1 μm in diameter, covalently bound to carboxyl acid, were purchased from Invitrogen (no. 650.11, Dynal Biotech). Beads were washed twice with 1 × TX buffer (50 mM KCl/20 mM Tris·HCl, pH 8.4) and then coupled with 5′-amino-modified oligonucleotide acting as forward primers (Table 1) according to the method described by Kojima *et al.* [17]. After binding, the beads were washed three times with 1 × TX buffer to thoroughly remove unbound oligonucleotides. The oil phase was composed of 4.5% Span 80 (no. S6760, Sigma) and 0.40% Tween 80 (no. S-8074, Sigma) in mineral oil (no. M-3516, Sigma). The aqueous phase contained 1 × Taq buffer (10 mM Tris–HCl, pH 8.0, 50 mM KCl), 3.5mM each dNTP, 25 mM MgCl2, 3 μM each reverse primer, 0.04 μM each forward primer, 0.5 U/μL Ex Taq (TaKaRa) and 10 ng/μL sample DNA.

Water-in-oil emulsions were prepared by dropwise addition of 1,000 μL of the aqueous phase to 2,000 μL of the oil phase previously placed in an IKA tube (45-mL round-bottom cryogenic vial). The dropwise addition was performed over ≈1 min while the mixture stirred at 600 rpm with an ULTRA-TURRAX® Tube Drive (IKA). After the addition of the aqueous phase, the mixture continued to be stirred for a total time of 5 min. The emulsions were aliquoted into a 96-well PCR plate, each contained about 100 μL. PCR was carried out under the following cycling conditions: 94 °C for 2 min, 45 cycles of 94 °C for 15 sec, 62 °C for 30 sec, and 70 °C for 45 sec.

### 3.5. Separation and Labeling of the Target Molecules

After PCR cycling, the emulsion from the 96 wells of a PCR plate were pooled and broken by the addition of twice the volume of 2-butanol and transferred to a 50 mL centrifugal tube. After vortexing for ≈20 sec, the beads were pelleted by centrifugation in a centrifuge at 500 ×g for 5 min. The top oil phase and all but ≈500 μL of the aqueous phase were removed from the tube, the aqueous phase was transferred to another clean tube, the beads were washed with NX buffer (100 mM NaCl/1% Triton X-100, 10 mM Tris·HCl, pH 7.5, and 1 mM EDTA) three more times and washed an additional three times with 1 × TX buffer by using magnetic separation rather than centrifugation. Onre hunderd μL of denaturing buffer was added and mixed thoroughly for about 2 min. Vortex 20 s. The beads were separated by magnetic separation, the supernatants collected, and cleanup with a Qiagen PCR purification kit was performed. Finally elution in 40 μL elute buffer was carried out. Cy3-labeled dUTP was added to 3-end of the target molecules by terminal transferase according to Rogers’s method [33].

### 3.6. Hybridization and Scanning

Fluorescently labeled target DNA were mixed with one volume of hybridization solution (10× SSC, 0.2% SDS, 50% formamide, 100 μg/mL sheared Salmon Sperm DNA) and heated to 95 °C for 3 min, then hybridized to capture DNA probes on a slide at 50 °C for 30 min. After hybridization, the slides were washed in 2× SSC (1× SSC is 0.15 mol/L NaCl plus 0.015 mol/L sodium citrate), 0.1% sodium dodecyl sulfate (SDS) at 50 °C for 5 min, 0.2 × SSC, 0.1% SDS, and distilled water at room temperature for 5 min in sequence and were then dried for scanning. All the glass slides were scanned on a LuxScan™ 10K system (CapitalBio Corporation), laser lights with a wavelength of 532 nm were used to excite Cy3 dye, and image analysis was carried out with QuantArray software.

## 4. Conclusions

In conclusion, emulsion and beads were used in this study to solve the serious problem of interferences between primers and template which commonly occurred in conventional multiplex PCR. A hybridization-based microarray technique was used to overcome the common disadvantages of electrophoresis. We successfully detected 13 toxin gene sites at one time, and genes related to zearalenone were detected from one randomly purchased maize sample by this emulsion-based microarray method. Our experiment results proved its reliability, sensitivity and efficiency and show its great potential for practical applications in both microbiological detection and academic research. 
